# Supplementary material for: Prevalence of post-traumatic stress disorder on health professionals in the era of COVID-19 pandemic, Northwest Ethiopia, 2020: A multi-centered cross-sectional study
Source: PLoS One. 2021 Sep 14;16(9):e0255340. doi: 10.1371/journal.pone.0255340 (PMC8439479; doi:10.1371/journal.pone.0255340)
Supplement: S1 File — (DOCX) [file pone.0255340.s001.docx]

# Annexes

## 1.1 Annex I: English version consent form

### 1.1.1 Participants information Document

Code No---------------

Date-------------------

Dear participants.

My name is ________________; I am a data collector for the research which is conducted by Sintayehu Asnakew, Getasew Legas Molla, Tewachew Muche, Amsalu Belete, Kalkidan Haile^,^ Getachew Yideg Yitbarek, Wubet Alebachew Bayih, Dejen Getaneh Feleke, Biniam Minuye Birhan, Haile Amha Yitbarek, Shegaye Shumet, and Ermias Sisay Chanie who are instructors at DTU. The purpose of the research is to assess the prevalence of post-traumatic stress disorder on health professionals in the era of COVID-19 pandemic, Northwest Ethiopia, 2020: multi-centred cross-sectional study

This will be critical input for health managers involved in mental health program. Your participation in this research is voluntary. If you decide not to participate there will be no negative consequences and no benefits for you. Except, if you have scored high on the measurement scale, with your permission, be referred to any hospital that have mental health services for assistance. However, your participation on this study is very important for achievement of the study and for paying the way to develop evidence based mental health promotion, disease prevention and treatment for the integration of mental health service in the early detection and giving appropriate care for HCWs thereby increasing the quality of care for these people. All the responses given by you and results obtained will be kept confidential using coding system whereby no one will have access to your response. You are not expected to give your name or phone number. Without permission from you and legal body any part of this study will not be disclosed to third person. You have full right to refuse and withdrawal to participate in this study if you don’t wish at any time.

The Interview period will take about 15 minutes. If you are willing to participate in this study, you need to understand and sign the agreement form and then you will give your responses to data collectors.

### 1.1.2. Informed consent form

I confirm that I understand the contents of this document and the nature of the research project, and I consent to participate voluntarily in the research project. I understand that I have autonomy to withdraw from the project at any time.

Signature of Participant………………………………......................Date ........................

Name and signature of supervisor…………………........................Date..........................

Name and signature of data collector ……………………...............Date........................

**1. Sociodemographic factors**

| No | Questions? | Alternative response | Code |
| --- | --- | --- | --- |
| 1 | What is your age in year? | ­­­­­­­­ ………………………………………… |  |
| 2 | Sex | 1. Female 2. Male |  |
| 3 | Marital status | 1. Married 2. Divorced 3. Single 4. Separated 5. Widowed |  |
| 4 | Ethnicity | 1. Amhara 2. Tigray 3. Oromo 4. Gurage 5. Other, ---------------------------------------------- |  |
| 5 | Religion | 1. Orthodox 2. Catholic 3. Muslim. 4. Protestant 5. Other ---------------------------- |  |
| 6. | profession | 1. Nurse 2. Doctor 3. Laboratory 4. Pharmacist 5. Midwifery 6. Other ------------------------------------------------- |  |
| 7 | Educational status | 1. Diploma 2. Degree 3. Msc 4. Specialist |  |
| 8 | Having Children | 1. Yes 2. No |  |

**2. Clinical and service factors**

8. Does your hospital have adequate and standardized PPE supply for compacting COVID-19 pandemic?

1. Yes
2. No

9. Do you have known medical problems like DM, asthma etc?

1. Yes
2. No

10. Do you have families who have chronic illness like DM, asthma?

1. Yes
2. No

11. Have you ever been diagnose with mental illness and treated previously

1. Yes
2. No

12. Do you feel as you are stigmatized because you are health professional in relation to COVID-19 pandemic by the community? (Like refused access to public transport, evicted from rented homes).

1. Yes I felt stigmatized
2. No I did not feel stigmatized

3. **Social support questions**

| **No.** | **Questionnaire** | **Alternative response** | **Code** |
| --- | --- | --- | --- |
| **11** | How many people are you so close to that you can count on them if you have great personal problems? | 1. None 2. 2. 1-2 3. 3. 3-5 4. 5 and above |  |
| **12** | How much interest and concern do people show in what you do? | 1. Very little 2. Little 3. Uncertain 4. Some 5. A lot |  |
| **13** | How easy is it to get practical help from neighbors if you should need it? | 1. Very difficult 2. Difficult 3. Possible 4. Easy 5. Very easy |  |

**4. IER-22 questions used to measure PTSD**

| SNo | questions | | Response | | | | | | | | |
| --- | --- | --- | --- | --- | --- | --- | --- | --- | --- | --- | --- |
|  |  |  | 1.Not at all | | 2.A little bit | | 3.Moderately | | 4.Quit a bit | | 5.Extremely |
|  | Intrusion | | | | | | | | | | |
| 1 | Any reminder brought back feelings about it. | |  | |  | |  | |  | |  |
| 2 | I had trouble staying asleep. | |  | |  | |  | |  | |  |
| 3 | Other things kept making me think about it. | |  | |  | |  | |  | |  |
| 4 | I thought about it when I didn’t mean to. | |  | |  | |  | |  | |  |
| 5 | Pictures about it popped into my mind. | |  | |  | |  | |  | |  |
| 6 | I found myself acting or feeling like I was back at that time. | |  | |  | |  | |  | |  |
| 7 | I had waves of strong feelings about it. | |  | |  | |  | |  | |  |
| 8 | I had dreams about it. | |  | |  | |  | |  | |  |
|  | Avoidance | | | | | | | | | | |
| 9 | I avoided letting myself get upset when I thought about it or was reminded of it. | |  |  | |  | |  | |  | |
| 10 | I felt as if it hadn’t happened or wasn’t real. | |  |  | |  | |  | |  | |
| 11 | I stayed away from reminders about it. | |  |  | |  | |  | |  | |
| 12 | I tried not to think about it. | |  |  | |  | |  | |  | |
| 13 | I was aware that I still had a lot of feelings about it, but I didn’t deal with them. | |  |  | |  | |  | |  | |
| 14 | My feelings about it were kind of numb. | |  |  | |  | |  | |  | |
| 15 | I tried to remove it from my memory. | |  |  | |  | |  | |  | |
| 16 | I tried not to talk about it. | |  |  | |  | |  | |  | |
| Hyper arousal | | | | | | | | | | | |
| 17 | | I felt irritable and angry. |  |  | |  | |  | |  | |
| 18 | | I was jumpy and easily startled. |  |  | |  | |  | |  | |
| 19 | | I had trouble falling asleep. |  |  | |  | |  | |  | |
| 20 | | I had trouble concentrating. |  |  | |  | |  | |  | |
| 21 | | Remember of it caused me to have physical reactions ,such as sweating trouble breathing ,nausea ,or pounding heart |  |  | |  | |  | |  | |
| 22 | | I felt watchful and on guard |  |  | |  | |  | |  | |

# 1.አኔክስ

## አኔክስ 1. 1. የአማርኛ መጠይቅ

### 1.1.1.የተሳታፊ መረጃ ቅጽ እና ተሳትፎ ማረጋገጫ

**ውድተሳታፊዎች**

ስሜ............................ይባላል፡፡በደብረታቦር ዩኒቨርሲቲ መምህር ለሆኑት መምህር ስንታየሁ አስናቀዉ፣ጌታሰዉ ለጋስ፣ተዋቸዉ ሙጬ፣አምሳሉ በለጠ፣ቃልኪዳን ሀይሌ፣ጌታቸዉ ይደግ ይትባረክ፣ዉበት አለባቸዉ ባይህ፣ደጀን ጌታነህ ፈለቀ፣ብንያም ምንዉየ ብርሃን፣ሀይሌ አምሀ የትባረክ፣ሸጋየ ሹመት፣እና ኤርሚያስ ሲሳይ ጫኔ ለሚሰሩት ምርምር መረጃ ሰብሳቢ ነኝ፡፡

ይህ ቅጽ በዚህ ምርምር ላይ ተሳታፊ እንዲሆኑ ለመጋበዝ ሲሆን የምርምሩ ዓላማም የኮሮና ቫይረስ በጤና ባለሞያዎች ላይ የሚያደርሰዉን የድህረ አደጋ የአዕምሮ መታወክ እና ለነዚህ ችግሮች ሊያጋልጡ የሚቸሉ ምክኒያቶችን ማጥናት ነው። ይህ ጥናት ከስነ-ዓዕምሮ ጤና ጋር በተያያዘ ለሚሰሩ ተቋማት ከፍተኛ ጠቀሜታ ይኖረዋል ፡፡ በዚህ ጥናት ለመሳተፍ ዉሳኔዉ የእርሰዎ ነዉ ፡፡ መሳተፍ ካልፈለጉም በእርሶዎ ላይ የሚመጣብዎ ምንም ዓይነት ችግር አይኖርም ፡፡ ለመሳተፍ ከወሰኑም ምንም ዓይነት ጥቅም አይኖሮትም ሆኖም ግን በምርመሩ መስፈርት ህክምና አስፈላጊ ሆኖ ከተገኘ በእርሶዎ ፈቃድ ወደ ህክምና ተመርተዉ እገዛ ያገኛሉ፡፡ በዚህ ጥናት ላይ የሚሰጡ ማንኛዉም አስተያየት እና መልስ በኮድ ስርዓት በሚስጢራዊነት የሚቀመጥ ሲሆን ለማንም ሰዉ አይሰጥም፡፡ ሰምዎንም ሆነ ስልክ ቁጥርዎን መስጠት አይጠበቅቦዎትም፡፡ ቃለመጠይቁ 15 ደቂቃ የሚፈጅ ሲሆን በዚህ ጥናት ላይ ለመሳተፍ ከፈቀዱ የስምምነት ፊርማ በቅጹ ላይ መፈረም ይኖርበዎታል፡፡ ከዚያም በመረጃ ሰብሰቢዎች ምላሸዎን እነዲሰጡ ይጠየቃሉ፡፡

ቃለመ ጠይቁ ላይ ለመሳተፍ ፈቃደኛ ነዎት ? 1. አዎ 2. አይደለሁ

**የተሳታፊ ማረጋገጫ**

የሰነዱን ይዘት የተረዳሁ ሲሆን የምርምር ፕሮጄክቱንም ዓላማ ተረድችያለሁ ፡፡ በዚህ ምርምር ፕሮጄክት ላይም ለመሳተፍ ፈቃደኛ ሆኛለሁ ፡፡ በማንኛዉም ሰዓትም ከጥናቱ ራሴን ለማግለል መብት እንዳለኝ አዉቂያለሁ፡፡

የተሳታፊ፣ፊርማ……………………………………….. …... ……….. ቀን……………..

የመረጃ ሰብሳቢ ስም ናፊርማ…………………………………………….. ቀን………………

የሱፐርቫይዘር፤ስምናፊርማ……………………………………………….....ቀን……………

ክፍል I:- የማህበራዊ መጠይቅ

| ተ.ቁ | መጠይቅ | ኣማራጮች |
| --- | --- | --- |
| 1 | እድሜዎ ስንት ነው | ­­­­­­­­ ………………………………………… |
| 2 | ጾታ | 1. ሴት 2. ወንድ |
| 3 | የጋብቻ ሁኔታ | 1. ያገባ /ች 2. የተፋታ/ች 3. ያላገባ/ጽ 4. ተለያይተዉ የሚኖሩ 5. የሞተበት/ባት |
| 4 | ብሄር | 1. አማራ  2.ትግሬ  3.ኦሮሞ  4.ጉራጌ  5. ሌላ, ---------------------------------------------- |
| 5 | ሀይማኖት | 1. ኦርቶዶክስ 2. ካቶሊክ 3. ሙስሊም 4. ፕሮቴስታንት 5. ሌላ ---------------------------- |
| 6 | ሞያ | 1. ነርስ 2. ሀኪም 3. ላቦራቶሪ 4. ፋርማሲስት 5. ሚድዋይፍሪ 6. ሌላ------------------------------------------------- |
| 7 | የትምህርት ደረጃ | 1. ዲፕሎማ 2. የመጀመሪያ ዲግሪ 3. ሁለተኛ ዲግሪ 4. ስፔሻሊስት |
| 8 | ልጅ አለዎት | 1. አለኝ 2. የለኝም |

**2. ክሊኒካል እና ስለአገልግሎት መጠይቅ**

8. ሆስፒታላችሁ ኮሮናን ለማከም በቂ እና ደረጃውን የጠበቀ እራስን ከኮሮና ለመከላከል የሚጠቅሙ ማቴሪያሎችን (PPE) አቅርቦላችሗልን?

1. አዎ
2. የለም

9. የታውቀ የውስጥ ደዌ ህመም እንደ አስም፣የልብ ድካም፣ ስኳር ወዘተ አለበህ/ሽ?

1. አዎ
2. የለብኝም

10. ከቢተሰብህ/ሽ ውስጥ የታውቀ የውስጥ ደዌ ህመም እንደ አስም፣የልብ ድካም፣ ስኳር ወዘተ ያልበት ሰው አለ?

1. አለ
2. የለም

11. የታወቀ የአዕምሮ ህመም ኣለብህ/ሽ

1. አዎ
2. የለብኝም

12. እርስዎ የጤና ባለሞያ በመሆኔ ከኮሮና ጋር በተያያዝ መገለል ደርሶብኛል ይላሉ? ( ለምሳሌ ከትራንስፖርት መከልከል ፣ ከቤት ኪራይ መባረር ወዘተ)

1. ኣዎ ደርሶብኛል
2. አልደረሰብኝም

**3. ማህበራዊ ድጋፍን የሚመለከቱ ጥያቄዎች**

| ተ.ቁ | መጠይቅ | ምርጫ | ኮድ |
| --- | --- | --- | --- |
| 1 | በህይወትዎ ውስጥ በጣም ቅርብ የሆኑ እና ችግሮችን የሚካፈሉ ስንት ሰዎች አሉ? | 1. ምንም የለም 2. 1 - 2 3. ከ3-5 4. ከ5 በላይ |  |
| 2 | ሌሎች ሰዎች እርስዎ በሚያደርጓቸው ነገሮች ላይ ምን ያህል ግድ ያላቸው ና የሚያስብልዎት ይመስልዎታል | 1. ጨርሶ ግድ የላቸውም 2. ብዙም ግድ የላቸውም 3. እርግጠኛ መሆን አልችልም 4. በመጠኑ ግድ አላቸው 5. በጣም ግድ አላቸው |  |
| 3 | ከጎረቤቶዎ እርዳታ ማግኘት ምን ያህል ቀላል ነው | 1. በጣም ከባድ ነው 2. ከባድ ነው 3. ቀላል ባይሆንም እርዳታ ማግኘት ይቻላል 4. ቀላል ነው 5. በጣም ቀላል ነው |  |

**4. IER-22 - የድህረ አደጋ የስሜት መረበሽን መለካት (ፒቲስዲ)**

| ተቁ | መጠይቆች | ምላሽ | | | | |
| --- | --- | --- | --- | --- | --- | --- |
|  |  | 1.በጭራሽ | 2.ትንሽ | 3.በመጠኑ | 4.በጣም | 5.እጅግ በጣም |
| 1 | ስለኮሮና ማንኛውም አስታዋሽ ነገር ሲኖር ስሜቴን ልክ እንደመጀመሪያው ይመልሰኛል |  |  |  |  |  |
| 2 | ለመተኛት ችግር ነበረብኝ |  |  |  |  |  |
| 3 | ሌሎች ነገሮች ስለኮሮና ጉዳይ እንዳስብ ያደርጉኛል ፡፡ |  |  |  |  |  |
| 4 | ሳልፈልግ ስለኮሮና አስባለሁ |  |  |  |  |  |
| 5 | ስለ ኮሮና የሚገልጹ ሥዕሎች ኮሮና በአእምሮዬ ውስጥ እንዳይወጣ እያደረገኝ ኘው |  |  |  |  |  |
| 6 | አንዳንዴ እራሴን ልክ በመጀመሪያ ኮሮና እንደገባ አይነት ስሜት ውስጥ እንዳለሁ ይሰማኛል |  |  |  |  |  |
| 7 | በኮሮና ምክኒያት በከፍተኛ ስሜት ተጽዕኖ ውስጥ ነበርኩ |  |  |  |  |  |
| 8 | ስለ ኮሮና ያሉ ሁኔታዎች በህልሜ ይታዩኝ ነበር እየታዩኝም ነው ፡፡ |  |  |  |  |  |
| 9 | ስለኮርና ሳስታውስ እንዳልረበሽ እራሴን ለማራቅ ጥረት አደርጋለሁ |  |  |  |  |  |
| 10 | አንዳንዴ ኮሮና ያልተከሰተ እና እውነት ያልሆነ ይመስለኛል |  |  |  |  |  |
| 11 | ስለ ኮሮና ጉዳይ ከሚያስታውሱኝ ነገሮች እርቃለሁ . |  |  |  |  |  |
| 12 | ስለ ኮሮና ላለማስታዎስ ጥረት ኣደርጋለሁ |  |  |  |  |  |
| 13 | ስለኮሮና ብዙ የተደበላለቁ ስሜቶች እንዳሉኝ እገነዘባለሁ፣ግን ለመፍታት ጥረት ኣላደርግም |  |  |  |  |  |
| 14 | አንዳንዴ ስለኮሮና ስሜቴ ይጠፋብኛል ወይም ደብዛዛ ስሜት ይኖረኛል |  |  |  |  |  |
| 15 | ኮሮናን ከትውስታየ ሙሉበሙሉ ለማስወገድ እሞክራለሁ |  |  |  |  |  |
| 16 | ስለ ኮሮና ላለማውራት እሞክራለሁ |  |  |  |  |  |
| 17 | የመናደድ እና የመበሳጨት ስሜት ይሰማኛል |  |  |  |  |  |
| 18 | በቀላሉ የመበርገግ እና የመደንገጥ ስሜት ይሰማኛል. |  |  |  |  |  |
| 19 | ለመተኛት መቸገር ነበረብኝ ኣለብኝም. |  |  |  |  |  |
| 20 | ለነገሮች ትኩረት ማድረግ መቸገር አለብኝ |  |  |  |  |  |
| 21 | ስለኮሮና ሳስታውስ አካላዊ ግብረ መልሶች እንደ ላብላብ ማለት፣ የመተንፈስ ችግር ፣ ማቅለሽለሽ የልብ ምት መጨመር ይታይብኛል |  |  |  |  |  |
| 22 | ንቁ መሆን ነገገሮችን በንቃት መከታተል ሁሌም ዝግጁ መሆን አይነት ስሜት ይሰማኛል |  |  |  |  |  |
