# Supplementary material for: Prevalence of post-traumatic stress disorder on health professionals in the era of COVID-19 pandemic, Northwest Ethiopia, 2020: A multi-centered cross-sectional study
Source: PLoS One. 2021 Sep 14;16(9):e0255340. doi: 10.1371/journal.pone.0255340 (PMC8439479; doi:10.1371/journal.pone.0255340)
Supplement: Attachment — Submitted filename: Response to Reviewers.docx [file pone.0255340.s002.docx]

**Reviewers’ comments and authors response**

After going through the entire manuscript, you forwarded your constructive comments which have been missed to touch. Therefore, we are glad enough to express our sincerest thanks for your constructive editorial comments that could help improve novelty of our work. All the comments that have been included are highlighted in the track changes

**Editors’ comment and suggestions(1)**

1. Please ensure that your manuscript meets PLOS ONE's style requirements, including those for file naming.

**Authors Response**

Thank you very much, we got these comments with paramount significant and we tried to rewrite the manuscript using PLOS ONE's style requirements per the academic editors’ suggestion

**Editors’ comment and suggestions(2)**

2. Please include in your Methods section (or in Supplementary Information files) the participating hospitals/institutions. Furthermore, please include additional information regarding the survey or questionnaire used in the study and ensure that you have provided sufficient details that others could replicate the analyses. For instance, if you developed a questionnaire as part of this study and it is not under a copyright more restrictive than CC-BY, please include a copy, in both the original language and English, as Supporting Information./963.

**Authors Response**

Yes indeed! We authors included the participating hospitals /institutions in the method section and the questionnaire has been supplied as supporting file both in Amharic and English versions

**Editors’ comment and suggestions(3)**

3. Thank you for stating the following financial disclosure:

"he funders had no role in study design, data collection and analysis, decision to publish, or preparation of the manuscript."

At this time, please address the following queries:

a. Please clarify the sources of funding (financial or material support) for your study. List the grants or organizations that supported your study, including funding received from your institution.
b. State what role the funders took in the study. If the funders had no role in your study, please state: “The funders had no role in study design, data collection and analysis, decision to publish, or preparation of the manuscript.”
c. If any authors received a salary from any of your funders, please state which authors and which funders.
d. If you did not receive any funding for this study, please state: “The authors received no specific funding for this work.”

Please include your amended statements within your cover letter; we will change the online submission form on your behalf.

**Authors Response**

Since the authors did not receive any fund for this work, we included in the previous submitted form but to make it in PLOS ONE's writing format, we amended and included in the cover letter as “The authors received no specific funding for this work”.

**Editors’ comment and suggestions (4)**

4. Your ethics statement should only appear in the Methods section of your manuscript. If your ethics statement is written in any section besides the Methods, please move it to the Methods section and delete it from any other section. Please ensure that your ethics statement is included in your manuscript, as the ethics statement entered into the online submission form will not be published alongside your manuscript.

**Authors Response**

Yes indeed! The ethical statement was written in both declaration and methods section so that we deleted the part in the declaration section and only included in the method section per the editors’ comment and suggestion.

**Editors’ comment and suggestions (5)**

5. Thank you for submitting the above manuscript to PLOS ONE. During our internal evaluation of the manuscript, we found significant text overlap between your submission and the following previously published works, some of which you are an author.

**Authors Response**

- **Great thanks!** The whole manuscript was edited by language expert and the changes are included throughout the revised version of the manuscript and the whole manuscript has been rephrased to remove the duplicated text.

**Review Comments to the Author**

**Reviewer #1:**

This manuscript intended to reveal the prevalence and predictors of PTSD in health professionals, which addressed an important question. However, the current version suffered from several problems.

**Reviewer s’ comment and suggestions (1)**

1. In introduction, predictors of PTSD under COVID-19 should be addressed with latest literature.

**Authors Response**

Yes indeed! This is the part that has been missed. Thus, we have intensively searched the previous published literatures and those factors which predicted PTSD has been included in the revised manuscript.

**Reviewer s’ comment and suggestions (2)**

2. More information was expected about the participants: e.g., educational level, department.

**Authors Response**

Yes, we authors missed the details especially about the educational status of the participants and other socidempgrpahic characteristics and now their detail is included in the revised manuscript.

**Reviewer s’ comment and suggestions (3)**

3. 423 was calculated as sufficient sample size. However, only 396 participants were included finally, which was insufficient. How is the process of recruitment of participants? Why not more people were invited initially?

**Authors Response**

In this study there are 8 participating hospitals with a total of 736 health professionals

We proportionally allocated the sample size to each hospital and we invited 423 participants by using simple random sampling technique. Of these, twelve (12) of the eligible participants refused to participate and five (5) of the questionnaire were discarded because of incomplete data. Finally, 396 questionnaires were included for the analysis. That is why we included the 10% percent non-response rate to the initially calculated sample size.

**Reviewer s’ comment and suggestions (4)**

4. The instrument Oslo needs more information, such as validity, sample question, et al.

**Authors Response**

Social support was measured by the Oslo 3-item Social Support Scale. The social support scores ranges from 3-14 , in that scores from 3-8 as poor,9-11 as moderate and 12-14 as strong social support but for this research purpose it was categorized into poor for scores less than nine . The scores from 9–14 were considered moderate to strong support and merged together as social support. The OSSS-3 is the three items questionnaire assessing the possibility of social support. The three questions assessing social support in OSSS-3 are:

1. How many people are you so close to that you can count on them if you have great personal problems?

2. How much interest and concern do people show in what you do?

3. How easy is it to get practical help from neighbors if you should need it?

The Oslo Social Support Scale has been used in population level studies in Ethiopia and showed good predictive value.

**Reviewer’s comment and suggestions (5)**

5. In regression, I wonder why not all variables were included in the analysis such as age, gender, and profession? Maybe these variables predicted PTSD significantly.

**Authors Response**

Yes indeed! all variables including socio-demographic factors (age, gender, profession, marital status, educational status, and having children), clinical variables (family history of medical illness, history of mental illness, having medical illness, psychosocial and material factors consists social support, perceived stigma and lack of adequate and standardized PPE supply were included in the bivariate analysis. However, only divorced, poor social support, not getting standardized PPE, having medical problems, history of mental illness, having families with medical health problems and perceived stigma were found to be significant with PTSD in the bivariate analysis. These variables were interred into multivariate logistic regression for further analysis to control confounding factors. Finally, perceived stigma, lack of standardized and adequate PPE supply, poor social support, having history of medical and mental illness were significantly associated with PTSD.

**Reviewer s’ comment and suggestions (6)**

6. Very limited predictors were analyzed in the current survey, in which other potentially important factors were not discussed, such as coping style, emotional status. Authors should list this in limitations at least.

**Authors Response**

Great look! We got this comment with paramount significance i.e. not only coping style, emotional status but also training about COVID-19 has been missed that should be included as factors. So we included in the limitation part which can for possible indication of other interested research in this area

**Reviewer s’ comment and suggestions (7)**

7. Overall, authors should pay more attention on the writing, including space, capitalization, punctuation, et al.

**Authors Response**

We have not any doubt with this comment. Hence from repeated proof-reading of the whole manuscript, we found several grammatical errors, interlinings, punctuation errors, wording and spelling errors. Thus, the whole manuscript was edited by language expert and the changes are included throughout the revised version of the manuscript.

**Review Comments to the Author**

**Reviewer #2**: Thank you for the opportunity to review this paper. It highlights the prevalence of PTSD among health care workers responding to the COVID-19 pandemic in Amhara, Ethiopia, and also points out risk factors (e.g., access to PPE, social support, medical problems) associated with PTSD. This is a useful addition to the growing literature on COVID-19. In order to get the message across effectively, I recommend heavily proofreading with an eye for English sentence structure and grammar. In addition, I have some comments about the analysis:
**Reviewer‘s comment and suggestions (1)**

**Major comments on analysis:**

1. What variables were in the multivariate logistic regression? Were demographic characteristics included? Things that affect the exposure and also affect PTSD should be included. I think it would be important to control for age, sex, marital status, profession, and perhaps some others, if possible. Whether to include all of the risk factors of interest in a single model should also be carefully considered. For example, perhaps social support should not be included in the assessment of marital status, as it may be a mediator of the effect of marital status on PTSD. Regardless, please indicate details about the variables in the model in both the text and a footnote to Table 3.

**Authors Response**

Yes indeed! all variables including socio-demographic factors (age, gender, profession, marital status, educational status, and having children), clinical variables (family history of medical illness, history of mental illness, having medical illness, psychosocial and material factors consists social support, perceived stigma and lack of adequate and standardized PPE supply were included in the bivariate analysis. However, only divorced, poor social support, not getting standardized PPE, having medical problems, history of mental illness, having families with medical health problems and perceived stigma were found to be significant with PTSD in the bivariate analysis. Furthermore those variables significantly associated with PTSD in the bivariate analysis were entered into the multivariate logistic regression to control the confounding variables. All these has been included in the text and table form in the revised manuscript

**Reviewer‘s comment and suggestions (2)**

2. “Social support = strong” only has 11 people with PTSD. This makes it a poor reference group and perhaps its use leads to the very large confidence intervals for the social support estimates. I recommend combining the “moderate” and “strong” categories, and making this the reference group

**Authors Response**

Great thanks! We authors took very important lesson and we made the corrections per the reviewer’s recommendation that is we combined moderate and strong social support in to one and made it the reference group.

**Reviewer‘s comment and suggestions (3)**

3. Please report either in the Methods section, or as the first comment in the Results section (not later) how many people were invited to participate and how many actually participated. In addition, please state whether people who chose not to participate were different than people who did participate in terms of key demographic characteristics.

**Authors Response**

In this study there were 8 participating hospitals with a total of 736 health professionals.

We invited 423 participants by using simple random sampling technique by proportionally allocating the sample size to each hospital. Of these, twelve (12) of the eligible participants refused to participate and five (5) of the questionnaire were discarded because of incomplete data. Finally, 396 questionnaires were included for the analysis. Those individuals who were on annual leave and severally ill were excluded. This has been included in the methods and the result section

**Minor comments:**

**Reviewer‘s comment and suggestions (4)**

4. The abstract should clearly state that the aims of the paper were to assess the prevalence of PTSD in this population and to assess the association of certain risk factors with PTSD in this population.

**Authors Response**

Corrected per the reviewer suggestion

**Reviewer‘s comment and suggestions (5)**

5. I would be interested to see more discussion of the “perceived stigma” findings. What types of stigma have health care workers in the Amhara region faced as a result of COVID-19 (or generally)?

**Authors Response**

Sure! Stigma during the era of COVID-19 pandemic is too high as reported by the general public and this condition is even worse in case of health care providers. This is because the communities’ perception that the health care providers have close contact with the COVID-19 patents so that being far away from them by any means is recommended. In this study the participants (health professionals) were asked as “Do you perceived as you are stigmatized because you are health professional in relation to COVID-19? (Like refused access to public transport, being isolated from any social affairs, and even evicted from rented homes) and the response was yes/no. Thus significant numbers of health professionals 95(24%) were stigmatized by the public. This indicates the need to increase the communities awareness about COVID-19 and the appropriate prevention strategies instead of stigmatizing the frontline health care workers which in turn enhances the care of patients especially COVID-19 cases.

**Reviewer‘s comment and suggestions (6)**

6. Is the IES-R validated for use in this population (e.g., Northwestern Ethiopia or Ethiopia in general)?

Authors Response

Great thanks for your constructive comment you provided us. Actually IES-R is not validated in Ethiopia case in these populations as to our searching knowledge. In this study, we conducted a reliability analysis for the IES-R-22questionnaire (Amharic version) and that it had a high score (Cronbach’s α=0.92). Thus, we assumed as a good measure PTSD in this study. In fact, it would be acceptable if it was validated but we cannot do this because of time constraints and we put as a limitation to show the necessity of its validation in this population by other interested researchers.

**Reviewer‘s comment and suggestions (7)**

7. A“limitations”section should be added.

Authors Response

We added this section per the reviewer’s recommendation and suggestion as **Limitation**

**Reviewer‘s comment and suggestions (8)**
8. Be consistent with labeling the pandemic as “COVID-19” versus “Corona” or other labels.

Authors Response

With no doubt, we took the comment and we made correction as COVID-19 pandemic through the revised manuscript

**Reviewer‘s comment and suggestions (9)**
9. The underlying data do not appear to be in the manuscript, as stated.

Authors Response

Sure! All the data can be accessible with reasonable request from the corresponding author
