# Supplementary material for: Prevalence of post-traumatic stress disorder on health professionals in the era of COVID-19 pandemic, Northwest Ethiopia, 2020: A multi-centered cross-sectional study
Source: PLoS One. 2021 Sep 14;16(9):e0255340. doi: 10.1371/journal.pone.0255340 (PMC8439479; doi:10.1371/journal.pone.0255340)
Supplement: Attachment — Submitted filename: Response to Reviewers 2nd.docx [file pone.0255340.s003.docx]

**Reviewers’ comments and authors response**

In the first place we would like to forward our great thanks for your constructive comments and suggestions for the improvement of the manuscript. Thus, all the comments that have been included are highlighted in the track changes.

Reviewer #1: The manuscript intended to reveal the prevalence of PTSD in health care professionals in Ethiopia during COVID-19 pandemic. The topic is interesting and deserves to explore. However, the writing of this version suffered from several major shortcomings.

Reviewer 1 comments and suggestions (1)

1. The English need to be improved by native speaker.

**Response**

Great thanks! We invited the language expert and the manuscript has been edited and the changes are included throughout the revised version of the manuscript.

Reviewer 1 comments and suggestions (2)

2. The relationship between IES and PTSD need more exploration in introduction.

**Response**

The relationships between IES and PTSD have been stated per the reviewer’s suggestion and comment

Reviewer 1 comments and suggestions (3)

3. The significance of this investigation to the current pandemic need to be clearly addressed in the introduction.

**Response**

Yes indeed! This is the part that we missed and correction has been made per the reviewer’s comments and suggestions

Reviewer 1 comments and suggestions (4)

4. How to health care professionals? This point need to be addressed in introduction or methods.

**Response**

We included this point in the introduction part per the reviewer’s suggestions and comments

Reviewer 1 comments and suggestions (5)

5. In introduction, latest literature referring to the PTSD in health care professionals especially during the current pandemic should be systematically reviewed.

**Response**

Certainly! We reviewed the newly published articles and included them in the introduction part.

Reviewer 1 comments and suggestions (6)

6. 423 participants are insufficient for the sample size, which indicated that the current sample size is in a low power of effect size. Any statistic methods carried out to improve the effect size? Or any other methods could be carried out to solve the problem?

**Response**

We want to say sorry for the confusion. In fact the total health professionals in the eight hospitals were 736. We then calculated the sample using single proportion formula giving the final sample size of 423and the response rate was 94.8%. Thus, the sample size, 423 is assumed to be sufficient for the study populations of 736 which could also be a representative

**Reviewer 1 comments and suggestions (7)**

7. The format of Figure and Table need to be attended, especially the capitalization and the accuracy of English (severe instead of sever)

**Response**

Yes indeed! We got the mistake and corrected in the revised figure

**Reviewer 1 comments and suggestions (8)**

8. Model information about the regression should be given in the results.

**Response**

Great thanks! This comment has been also raised by reviewer2 so that we revised the regression and put in the revised manuscript

Reviewer #2: Thank you for the opportunity to review the revised version of this manuscript. It is greatly improved. After the authors clarified the multivariate regression methods, I have do have some remaining concerns about that set of analyses.
Major comments:

Reviewer 2 comments and suggestions (1)
1. I am interested in seeing the bivariate results in addition to the multivariate results. These should all be displayed. I suggest one table with bivariate results, and another table with results of the multivariate analysis. Even the “non-significant” results should be shown, along with 95% CI. This would allow the reader to assess the size and precision of each association.

**Response**

Certainly! We revised the analysis and we put the analysis as bivariate and multivariate in separate tables in the revised manuscript

Reviewer 2 comments and suggestions (2)
2. In the multivariate analysis, I think that it is more important to choose variables to add in the multivariable model based on conceptual reasons/based on existing literature, rather than p<0.05 in the bivariate analyses. P>0.05 might be driven by small sample size or other statistical reasons. I think it would be particularly important to include age and gender in the multivariable model, since they are strongly associated with PTSD and many of the other factors in the model.

**Response**

Sure! We took the comments and suggestions given by the reviewer and we took all variables analyzed in bivariate into multivariate analysis. In this case, age was re categorized because in the cell there was 1.3 % frequency which was < 5%. Thus, age was significantly associated with PTSD.

Reviewer 2 comments and suggestions (3)
3. When interpreting results of the multivariable model, add “when controlling for the other variables in the model.” For example, “The odds of developing post-traumatic stress disorder among health care providers were 2.27 times higher among those participants who had not standardized PPE supply as compared with those who had standardized PPE supply, *controlling for the other variables in the model* (AOR=2.27,95CI;1.29,3.98).” Similarly, in the discussion, it should be made clear that the results described are from the multivariate (adjusted) models.

**Response**

Corrected per the reviewer suggestions and comments

Reviewer 2 comments and suggestions (4)
4. The following phrase on page 6 should be revise for clarity: “Factors associated with post-traumatic stress disorder were selected during the bivariate analysis with p<0.05 for further analysis in the multivariable logistic regression analysis. In the multivariable logistic regression analysis, variables with p<0.05 at 95% CI with adjusted OR were considered statistically significant.”

**Response**

Great thanks! we found it as confusing and correction has been made per reviewers suggestion

Reviewer 2 comments and suggestions (5)
5. I suspect that marital status is not significantly associated with PTSD in the multivariate model because social support is a mediator of this relationship (i.e., being unmarried leads to poor social support, which then leads to PTSD), and social support is controlled for. This does not have to be mentioned in the discussion, but could be.

**Response**

Certainly! We took great lesson here so that we checked multi-co linearity between independent variables including marital status and social support using VIF and tolerance. However, no multicolinearity was found between independent factors.

Minor comments:

Reviewer 2 comments and suggestions (1)
1. On page 3, several previous studies of the prevalence of PTSD are mentioned. Were these all among health care workers? Nurses, specifically? Please specify.

**Response**

Sure! The studies were among all health care workers

Reviewer 2 comments and suggestions (2)
2. Is South Gondar 666 *kilometers* from Addis Ababa? Miles? Please specify.

**Response**

Sorry for the confusion, it is kilometers and correction has been made on the revised manuscript

Reviewer 2 comments and suggestions (3)
3. In describing the sample size calculations, does P refer to prevalence? Also, I think that specifying Z=1.96, 95% CI, and alpha=0.05 is repetitive. I think just specifying a standard normal distribution and alpha=0.05 would be enough.

**Response**

Certainly! Redundancy of idea was noted and we corrected it per the reviewer’s suggestions and comments

Reviewer 2 comments and suggestions (4)
4. The sentence about the number of participants from each hospital could be rewritten as, “Health professionals were from Debretabor (N=325), Andabet (N=62), Estie (N=55)..." for clarity.

**Response**

Corrected per the reviewers comments and suggestions

Reviewer 2 comments and suggestions (5)
5. Please specify that 396 participants, not 396 questionnaires, were included.

**Response**

Corrected as participants in the revised manuscript

Reviewer 2 comments and suggestions (6)
6. It is noted that, “All health professionals working in South Gondar Zone hospitals were included, and those participants who were on annual leave and severely ill were excluded.” This is confusing following the statement that there were 396 participants included. Perhaps this comment should be moved to the beginning of the paragraph, if it is referring to initial eligibility criteria?

**Response**

Great look! It was really confusing, thus we made the necessary correction per the reviewers suggestions

Reviewer 2 comments and suggestions (7)
7. When describing the tools used to measure PTSD, social support, etc. the authors seems to discuss each tool, and then describe them all again in the same order. Can this section be restructured to have a single section on each tool?

**Response**

Sure! It was really redundant and confusing so that correction has been made by classifying as “data source and measurement” and operational definitions”

Reviewer 2 comments and suggestions (8)
8. In “data processing and analysis” please change “descriptive, bivariate, and multivariate logistic regression” to “descriptive statistics, and bivariate and multivariate logistic regression” (i.e., the word “statistics” is missing.)

**Response**

Great thanks! Correction has been made

Reviewer 2 comments and suggestions (9)
9. Page 9: State that the *prevalence*, not magnitude, of PTSD was 55.1%. Magnitude can be confused with severity.

**Response**

We replaced magnitude with prevalence per the reviewers comment

Reviewer 2 comments and suggestions (10)
10. Unless specified otherwise by editors, limitations may be better off before the conclusions.
 **Response**

We took the comment with paramount significance and we made it before the conclusion in the revised manuscript

Reviewer 2 comments and suggestions (11)
11. I suggest removing the detail about the number of participants invited from the abstract (and only leave the number that actually were included), as it is distracting from the primary message of the abstract. There is sufficient detail in the main manuscript.

**Response**

Great! Corrected per the reviewers comment and suggestions
