# Supplementary material for: Prevalence of post-traumatic stress disorder on health professionals in the era of COVID-19 pandemic, Northwest Ethiopia, 2020: A multi-centered cross-sectional study
Source: PLoS One. 2021 Sep 14;16(9):e0255340. doi: 10.1371/journal.pone.0255340 (PMC8439479; doi:10.1371/journal.pone.0255340)
Supplement: Attachment — Submitted filename: Response to Reviewers 3rd.docx [file pone.0255340.s004.docx]

**Reviewers’ comments and authors response**

In the first place we would like to forward our great thanks for your constructive comments and suggestions for the improvement of the manuscript. In addition, we would like to thank for scientific lesson we got throughout your comments and suggestions. Thus, all the comments that have been included are highlighted in the track changes.

Reviewer #1: Authors have done a good job in revision. I have only one suggestion: The language of this manuscript still needs improve, please improve the language substantially.

.

**Response**

Great thanks again! From repeated proof-reading, we found several grammatical errors, punctuations errors, wording and spelling errors etc. Therefore we invited an expert with Masters of Arts in English; we have tried our best to thoroughly copyedit the manuscript for appropriate English language usage. Thus the changes are found throughout the revised version of the manuscript.

Reviewer #2: Thank you for the opportunity to review a revised version of this manuscript. It is improved from the previous version. Thanks to the authors for adding the bivariate results. Although I list several comments below, they are minor in nature. I would also like to note that copy-editing is necessary.
Reviewer 2 comments and suggestions (1)
1. First sentence is confusing. I don’t think SARS should be mentioned, as it takes the focus away from COVID-19.

**Response**

Great thanks! Correction has been made per the reviewer’s suggestions

Reviewer 2 comments and suggestions (2)
2. In the first paragraph, “the election” is referenced. Which election?

**Response**

Great! It is the sixth national election of Ethiopia

Reviewer 2 comments and suggestions (3)
3. Information about IES-R was added to the introduction in this version. In my opinion, that should be saved for the methods section.

**Response**

Great thanks! We removed the information about IES-R in the introduction part and added in the method section per the reviewer’s suggestions and recommendations.
4. Fix the last sentence of the introduction: “magnitude and associated factors” is confusing. Maybe “prevalence of PTSD, as well as risk factors for PTSD.”

Reviewer 2 comments and suggestions (4)

**Response**
Yes! We replaced magnitude with Prevalence per the reviewer’s suggestions and comments

Reviewer 2 comments and suggestions (5)
5. I want to clarify point 2 in my previous review (“In the multivariate analysis, I think that it is more important to choose variables to add in the multivariable model based on conceptual reasons/based on existing literature, rather than p<0.05. P<0.05 might be driven by small sample size or other statistical reasons”). I think p<0.05 alone that this is not a good reason to choose variables for the multivariate model. The decision should also be based on existing literature. Perhaps you can say, “the bivariate results may have been subject to confounding. Therefore, we conducted a multivariate analysis containing all variables associated with PTSD. Variables for the multivariate model were selected based on a combination of known risk factors for PTSD from existing literature, and variables significantly associated with PTSD in the bivariate analyses.” Also, remove “not too large” from this section.

**Response**

Corrected per the suggestions and comments

Reviewer 2 comments and suggestions (6)
6. In Table 4, there should be a footnote stating that the results shown are adjusted for all of the other variables listed. I would also suggest adding, “when controlling for other variables in the model” to paragraph 2 in the Discussion. I think this information is important for interpreting the results from the multivariate (adjusted) model.

**Response**

Sure! Corrected per the reviewers suggestions and comments in the revised manuscript.

Reviewer 2 comments and suggestions (7)

7. Thank you for your revision in response to point 7 on my last review (“When describing the tools used to measure PTSD, social support, etc. the authors seems to discuss each tool, and then describe them all again in the same order. Can this section be restructured to have a single section on each tool?”). I appreciate that you have “data sources and measurement” and then “operational definitions.” It would still be my preference for easier reading to have each tool described once (measurement and operational definition together). As previously noted, some of this information is currently in the introduction, as well.
 **Response**

Great thanks! We made the necessary correction per the comments and suggestions in the revised manuscript.
Wording comments:

Reviewer 2 comments and suggestions (8)
8. Page 9: Instead of “found to be significant,” should be “found to be significantly associated with PTSD.

**Response**

Corrected per the recommendations of the reviewers

Reviewer 2 comments and suggestions (9)
9. On page 12, “magnitude” should be replaced with “prevalence.”

**Response**

Corrected per the reviewers recommendations

Reviewer 2 comments and suggestions (10)
10. On page 12, “corona outbreak” should be replaced with “COVID-19 pandemic.”

**Response**

Corrected per the reviewers recommendations
Reviewer 2 comments and suggestions (11)

11. On page 13, I am not sure what is meant by “conversely, being a physician affected PTSD positively.” Do

you mean “being a physician made PTSD less likely”?

**Response**

Certainly! It is really confusing and unclear sentences so that we made the corrections in the revised manuscript as “conversely physician were less affected by PTSD as compared with nurses”

Reviewer 2 comments and suggestions (12)
12. Replace “likely hood” with “likelihood” throughout.

**Response**

Corrected as “likelihood” throughout the revised manuscript
